# Relationship of Acylcarnitines to Myocardial Ischemic Remodeling and Clinical Manifestations in Chronic Heart Failure

**DOI:** 10.3390/jcdd10100438

**Published:** 2023-10-21

**Authors:** Yuri N. Belenkov, Anton A. Ageev, Maria V. Kozhevnikova, Natalia V. Khabarova, Anastasia V. Krivova, Ekaterina O. Korobkova, Ludmila V. Popova, Alexey V. Emelyanov, Svetlana A. Appolonova, Natalia E. Moskaleva, Ksenia M. Shestakova, Elena V. Privalova

**Affiliations:** 1 Hospital Therapy No. 1 Department, Federal State Autonomous Educational Institution of Higher Education I.M. Sechenov First Moscow State Medical University of the Ministry of Health of the Russian Federation (Sechenov University), 119435 Moscow, Russia; ageev_a_a@staff.sechenov.ru (A.A.A.); khabarova_n_v@staff.sechenov.ru (N.V.K.); krivova_a_v@staff.sechenov.ru (A.V.K.); korobkova_e_o@staff.sechenov.ru (E.O.K.); popova_l_v@staff.sechenov.ru (L.V.P.); emelyanov_a_v@staff.sechenov.ru (A.V.E.); privalova_e_v@staff.sechenov.ru (E.V.P.); 2 Laboratory of Pharmacokinetics and Metabolomic Analysis, Institute of Translational Medicine and Biotechnology, Federal State Autonomous Educational Institution of Higher Education I.M. Sechenov First Moscow State Medical University of the Ministry of Health of the Russian Federation (Sechenov University), 119435 Moscow, Russia; appolonova_s_a@staff.sechenov.ru (S.A.A.); moskaleva_n_e@staff.sechenov.ru (N.E.M.); shestakova_k_m@staff.sechenov.ru (K.M.S.)

**Keywords:** acylcarnitines, carnitines, metabolites, metabolism, myocardial remodeling, chronic heart failure, coronary heart disease

## Abstract

Background: Progressive myocardial remodeling (MR) in chronic heart failure (CHF) leads to aggravation of systolic dysfunction (SD) and clinical manifestations. Identification of metabolomic markers of these processes may help in the search for new therapeutic approaches aimed at achieving reversibility of MR and improving prognosis in patients with CHF. Methods: To determine the relationship between plasma acylcarnitine (ACs) levels, MR parameters and clinical characteristics, in patients with CHF of ischemic etiology (n = 79) and patients with coronary heart disease CHD (n = 19) targeted analysis of 30 ACs was performed by flow injection analysis mass spectrometry. Results: Significant differences between cohorts were found for the levels of 11 ACs. Significant positive correlations (r > 0.3) between the medium- and long-chain ACs (MCACs and LCACs) and symptoms (CHF NYHA functional class (FC); r = 0.31−0.39; *p* < 0.05); negative correlation (r = −0.31−0.34; *p* < 0.05) between C5-OH and FC was revealed. Positive correlations of MCACs and LCACs (r = 0.31−0.48; *p* < 0.05) with the left atrium size and volume, the right atrium volume, right ventricle, and the inferior vena cava sizes, as well as the pulmonary artery systolic pressure level were shown. A negative correlation between C18:1 and left ventricular ejection fraction (r = −0.31; *p* < 0.05) was found. However, a decrease in levels compared to referent values of ACs with medium and long chain lengths was 50% of the CHF-CHD cohort. Carnitine deficiency was found in 6% and acylcarnitine deficiency in 3% of all patients with chronic heart disease. Conclusions: ACs may be used in assessing the severity of the clinical manifestations and MR. ACs are an important locus to study in terms of altered metabolic pathways in patients with CHF of ischemic etiology and SD. Further larger prospective trials are warranted and needed to determine the potential benefits to treat patients with CV diseases with aberrate AC levels.

## 1. Introduction

Chronic heart failure (CHF) is the final stage of cardiovascular disease (CVD). Coronary heart disease (CHD) remains to be one of the main causes leading to the progression of CHF and development of left ventricular (LV) systolic dysfunction. In CHD, chronic or acute ischemia of cardiomyocytes (CMC) and associated inflammation, hypertrophy, and apoptosis of CMC underlie the formation of structural and functional myocardial remodeling (MR) [1]. At the biochemical level, these processes are caused by the inhibition of aerobic oxidation of the main energy substrate of CMC, fatty acids (FA), due to hypoxic damage of mitochondrial apparatus with the development of metabolic remodeling, represented primarily by activation of glycolysis and anaplerosis [2]. Under anaerobic conditions, there is an accumulation of oxidation intermediates toxic for CMC. These include underoxidized FAs determined in the carnitine-bound form—acylcarnitines (AC). Their endogenous pool consists mainly of carnitine, as well as short-, medium-, and long-chain ACs [3].

Several authors note that against the background of ischemia, prolonged inhibition of β-oxidation of FA in CMC, the deficiency of carnitine as a substrate for FA oxidation and, consequently, accumulation of AC initiates lipotoxicity and intracellular acidosis, contributing to the inhibition of energy production in CMC, increased oxidative stress, which leads to organelle degradation, CMC apoptosis, and eventually death of viable myocardium [4,5]. In this regard, it is assumed that circulating ACs reflect the development of structural and functional MR.

It is important to note that the severity of pathologic MR has a great influence on the further development of CHF of ischemic etiology [6]. At the same time, there is convincing evidence that progressive MR can be not only stopped, but also reversed, which results in improvement of both the systole-diastolic function (in particular, the restoration of the LV ejection fraction (LVEF) and the clinical condition of patients with CHF [7,8,9]). Since this process is important in determining the prognosis, course, and quality of further life in patients with CHF and CHD, it is necessary to identify potential markers of MR that can open new or help rethink the existing approaches in medication. We suggested that the level of circulating ACs may differ in patients with CAD and patients with CHF of ischemic etiology, and that these changes may be associated with myocardial remodeling and cardiac dysfunction. Clarification of the profile of altered levels of carnitine and its derivatives may allow, in the future, the development of targeted therapy for these metabolites, leading to an improvement in cardiac function in cardiac patients. Thus, the aim of the present study was to investigate the relationship of circulating plasma AC levels with structural and functional parameters of MR and clinical characteristics of patients with CHF of ischemic etiology and systolic dysfunction.

## 2. Materials and Methods

### 2.1. Study Design and Ethical Considerations

The prospective cohort study was conducted at the clinical bases of the Department of Hospital Therapy Sechenov University, Moscow, Russia, in the period from 2021 to 2022. The study included 2 comparison groups: the main group consisted of patients diagnosed with CHF of ischemic etiology and LVEF < 50% (CHF-CHD group); the control group included patients with CHD without signs of heart failure (CHD group). CHF was diagnosed according to current clinical recommendations [10], with determination of the N-terminal pro-brain natriuretic peptide (NT-proBNP) and using the clinical status assessment scale (NIHA) with further grading by the functional class (FC) of CHF. Patients were included in the main comparison group at the time of their hospitalization for decompensation of CHF. 

The diagnosis of CHD was established if patients had stenotic lesions of coronary arteries according to coronary angiography/computed tomographic coronary angiography and/or a history of myocardial infarction (MI) not earlier than 3 months before sampling for metabolite level estimation. Exclusion criteria were the following: Secondary arterial hypertension, cerebrovascular disorders (dementia; less than 6 months after acute cerebrovascular accident), acute renal failure, terminal renal failure (GFR < 15 mm/min/1.73 m^2^), signs and symptoms of liver disease in the decompensation stage, portal hypertension, uncontrolled bronchial asthma and chronic obstructive pulmonary disease, gastric or duodenal ulcer in the exacerbation stage, chronic pancreatitis in the exacerbation stage, malignant neoplasms, thyroid diseases, Cushing’s syndrome, type 1 diabetes mellitus, thrombocytopenia, hemorrhagic syndrome, autoimmune diseases, mental illness, alcoholism, drug addiction, substance abuse, pregnancy and breastfeeding.

Data of two-dimensional transthoracic echocardiography (TTE) were used to assess structural and functional changes in the heart. TTE was performed using commercial stationary equipment (TOSHIBA Aplio MX, Minato, Tokyo, Japan; SIEMENS Acuson SC2000, Berlin, Germany). Linear dimensions, chamber volumes (including indexed by body surface area), ventricular wall thickness, and cardiac functional parameters (LVEF; pulmonary artery systolic pressure (PASP)), as well as the dimensions of large vessels (inferior vena cava (IVC), pulmonary artery (PA), aorta) were evaluated according to the recommendations and considering sex differences.

The study was approved by the local ethical committee of Sechenov University and was conducted in accordance with the set of ethical principles for medical research involving human participants set out in the Declaration of Helsinki. Study participants were verbally informed of the detailed protocol and provided written informed consent to participate in this study. 

### 2.2. Laboratory Evaluations

Plasma samples were collected after an overnight fast between 8 and 10 am from the vein into vacuum tubes containing ethylenediaminetetetraacetic acid tricalic salt dehydrate. Immediately thereafter, samples were centrifuged at 2000 rpm for 20 min and stored at −80 °C until analysis. The MassChrom Amino Acids and Acylcarnitines Non-Derivatized 57.000 Kit (Chrom-systems, Munich, Germany) was used. Five μL aliquots of each plasma sample were mixed with 50 μL of a solution of isotopically labeled internal standards (ISTD) in methanol in a microtiter plate to precipitate proteins. After 10 min of incubation, the microtiter plate was centrifuged for 5 min at 100× *g*. Next, 40 μL of supernatant was transferred to the microtiter plate for analysis by flow injection using a tandem mass spectrometer (FIA-MS/MS). Analyses were performed in the positive electrospray ionization mode. Identification and quantification were achieved using multiple reaction monitoring (MRM).

Standardization was performed by spiking isotope-labeled standards into the samples. The FIA-MS/MS analysis was performed on a Waters TQ─S-micro system equipped with an electrospray ionization source (ESI) and coupled to a Waters Acquity I high-performance liquid chromatography (HPLC) pump (Waters Corp, Milford, USA). Flow injection analysis (FIA) was performed using a mixture consisting of 50% acetonitrile–water with 0.1% formic acid added at an isocratic flow rate of 200 μL/min. Mass spectrometry was performed under the following conditions: residence time 0.019–0.025 sec; capillary voltage 2 kV; nitrogen was used as the collision gas medium, and the source temperature was 150 °C. Target mass spectrometry data were imported and preprocessed using MassLynx software v4.1 (Waters, MA, USA). Metabolite concentrations were calculated from the signal intensities of analytes and corresponding internal standards. Samples for both comparison groups were analyzed in the same batch. The intra-assay precision for the kit used ranged from 4.9% to 7.6% and the inter-assay precision ranged from 7.2% to 17.2%. The error for metabolites ranged from −12.3% to 16.0%.

### 2.3. Statistical Analysis

The normality of data distribution was checked using the Shapiro–Wilk criterion. The mean (M) and standard deviation (SD) in the format of M ± SD, if the indicator had normal distribution, or the median (Me) and interquartile range [Q1; Q3] (in the format Me [Q1; Q3]) were used; otherwise, ANOVA Kruskal-Wallis with Bonferroni posterior analysis was employed to compare groups of more than two. To analyze the strength of the correlation relationship between the studied features, Pearson’s correlation coefficient (in case of normal distribution) and Spearman’s correlation coefficient (in case of non-normal distribution) were used. Statistical analysis was performed using the programs STATISTICA 12.0 and IBM SPSS Statistics 23.0.

## 3. Results

### 3.1. General Characteristics of the Groups

Ninety-eight patients were included in the study. Seventy-nine patients were included in the group of CHF-CHD, 19 patients in the group of CHD. General characteristics of the comparison groups are presented in Table 1. In terms of sex, age, and body mass index (BMI), the studied cohorts did not differ significantly. New note that BMI in the CHF-CHD group should be interpreted with correction for the presence of edema syndrome in patients, in connection with which the BMI value may be overestimated. At the same time, overweight or obesity of 1–2 degrees were determined in the CHD group.

Significantly more patients in the CHF-CHD group had a history of MI compared to the CHD group (73.4% vs. 27.8%, respectively). In the remaining patients who did not undergo MI, CHD was represented by proven coronary atherosclerosis with clinical manifestations of angina FC II-III according to Canadian classification. 

In contrast to patients in the CHF-CHD group in whom sinus rhythm was predominantly determined (73.7%), heart rhythm disorders (HRD) in the form of atrial fibrillation/atrial flutter (62%) of permanent form (25% of all HRDs) prevailed in the CHF- CHD group. In addition, four patients with CHF had implanted devices—pacemakers (Table 2).

**Figure 1:**
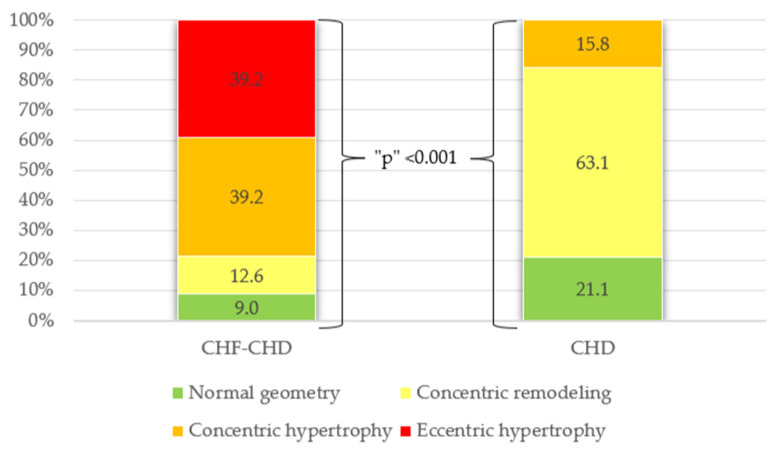
Distribution of patients by type of remodeling in the comparison groups. CHF—chronic heart failure; CHD—coronary heart disease; p - p-value.

Also, the comparison groups differed by the types of remodeling, which were determined by the ratio of LV myocardial mass index (LVMI, g/m^2^), and relative wall thickness (RWT): in patients with CHD, the leading types were concentric and eccentric hypertrophy, represented equally (39.2% each); in patients without signs of CHF, concentric remodeling prevailed (63.1%) (Figure 1).

### 3.2. Evaluation of Echocardiographic Parameters

The comparison groups differ naturally in LVEF and volume size indices of the left heart cavities—left ventricle (LV) and left atrium (LA)—which significantly exceed the upper limit of the normal and are statistically significantly higher in patients with CHF of ischemic etiology. The CHF-CHD group was characterized by the presence of signs of right heart overload—increased right atrial volume and LA parameters: its size and calculated left atrial systolic pressure (LASP), as well as significantly larger basal right ventricular diameter (RVD) compared to the CHD group, although its value remained within the acceptable normal range (Table 3).

### 3.3. Differences in AC Levels between Patients with CHF of Ischemic Etiology with LVEF <50% and Patients with CHD

**Figure 2:**
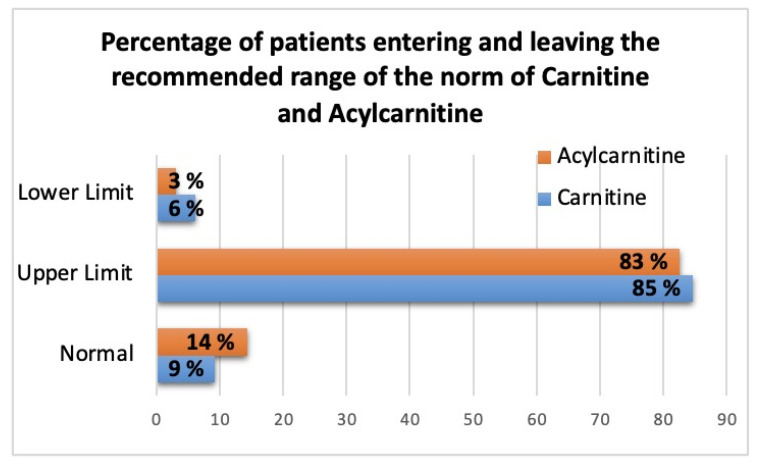
Prevalence of carnitine and acylcarnitine deficiency in patients with chronic heart disease.

**Figure 3:**
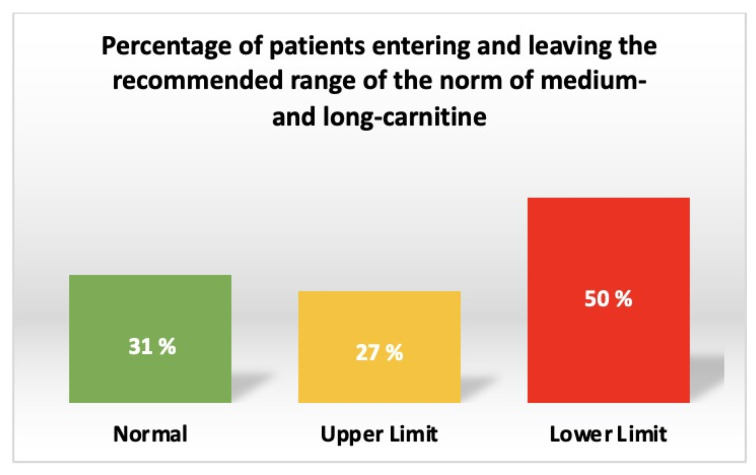
Prevalence of medium and long-chain acylcarnitine deficiency in patients with chronic heart disease.

Carnitine deficiency was found in 6% and acylcarnitine deficiency in 3% of all patients with chronic heart disease (Figure 2). At the same time, in 50% of all patients, the deficiency of medium- and long-chain acylcarnitines was determined (Figure 3).

To verify the differences in AC levels between the CHF-CHD group and the CHD group, their control pairwise comparison was performed. Significant differences (*p* < 0.05) were determined for 11 parameters, including those entered further into the correlation matrix (Table. 4): butyrylcarnitine (C4), decadienoylcarnitine (C10:2), dodecanoylcarnitine (C12), dodecanoylcarnitine (C12:1), tetradecanoylcarnitine (C14), tetradecanoylcarnitine (C14:1), tetradecadecadienoylcarnitine (C14:2), palmitoylcarnitine (C16), hexadecenoylcarnitine (C16:1), oleoylcarnitine (C18:1), linoleoylcarnitine (C18:2). According to these indices, the levels of all mentioned ACs were significantly higher in patients with CHF of ischemic etiology and LVEF <50% (Table 4). 

### 3.4. Relationship between AC Levels, Clinical Characteristics, and Remodeling Parameters in Patients with CHF of Ischemic Etiology with LVEF <50%

**Figure 4:**
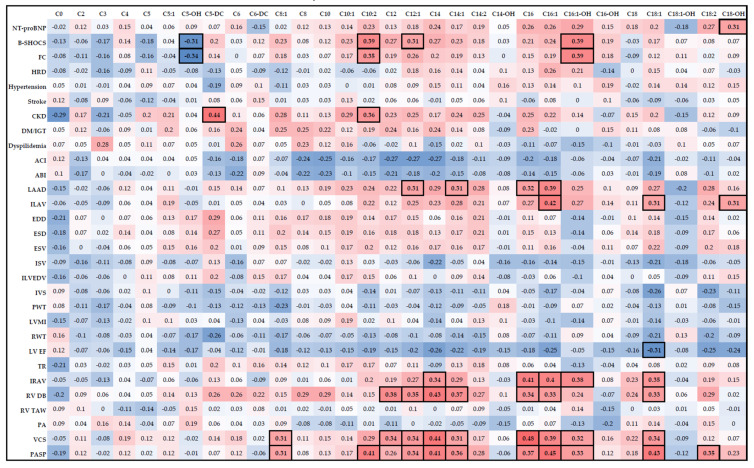
Correlation matrix with clinical and echocardiographic parameters of patients in the CCN-IBS group compared with acylcarnitine profile. Abbreviations: B-SHOCS, clinical status assessment scale (CSAS) score; FC—functional class; HRD—heart rhythm disturbances (AF); CKD—chronic kidney disease; DM—diabetes mellitus; IGT—impaired glucose tolerance; ACI—aortic root index; ABI—ascending aorta index; LAAD—left atrium anteroposterior dimension; ILAV—indexed LA volume; EDD—end-diastolic dimension; ESD—end-systolic dimension; ESV—end-systolic volume; ISV—indexed stroke volume; ILVEDV—indexed left ventricular (LV) end-diastolic volume; IVS—interventricular septum; PWT—posterior wall thickness; LVMI—LV myocardial mass index; RWT—relative LV wall thickness; LV EF—LV ejection fraction; TR–tricuspid regurgitation; IRA—indexed right atrial volume; RV DB—basal diameter of the right ventricle (RV); RV TAS—thickness of the anterior wall of the RV; PA—pulmonary artery size; VCS—size of the inferior vena cava; PASP—pulmonary artery systolic pressure. Comments are presented in the text. Significant correlations (*p* < 0.05) of moderate strength (r > 0.3 modulo) marked in black boxes, blue color means negative correlation, red color means positive correlation.

Correlations of moderate strength (r > 0.3 modulo) were revealed mainly between the level of AC with large and medium chain length and clinical characteristics of patients in the form of FC (Figure 4). Increased levels of medium- and long-chain (C10–C18) ACs were associated with a more severe course of CHF, pronounced signs of systolic congestion, which were the cause of significant exercise limitations (FC; r = 0.31−0.39; *p* < 0.05). In addition, decadienoylcarnitine (C10:2; r = 0.36; *p* < 0.05) and short-chain glutarylcarnitine (C5-DC; r = 0.44; *p* < 0.05) also showed an association with decreased renal function characteristic of this patient cohort. Hydroxyoctadecanoylcarnitine (C18-OH) correlated with NT-proBNP levels (r = 0.31; *p* < 0.05).

Also, the level of AC had a positive correlation of moderate strength with such echocardiographic parameters as LV size, volumes of both atria, dimensions of LV and vena cava inferior (Figure 2). The greatest number of correlations was found between the above types of AC and the level of pulmonary artery systolic pressure. Oleoylcarnitine showed negative correlation with LVEF (r = −0.31; *p* < 0.05), and hydroxyisovalerylcarnitine—with FC (r = −0.31−0.34; *p* < 0.05). 

**Figure 5:**
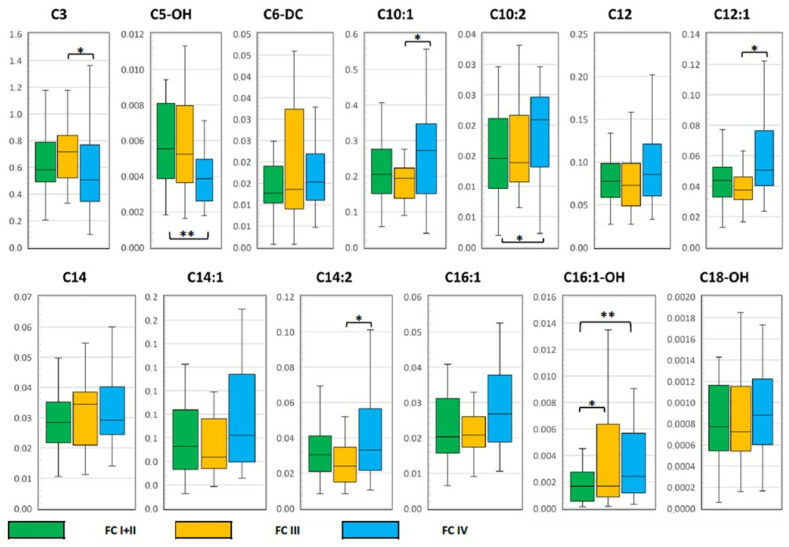
Box-plots based on the results of posterior analysis of AC levels as a function of SCOX FC within the CHF-CHD group. *—*p*-value <0.05; **—*p*-value <0.001. The units of measurement of the indicated AAs are μM. Comments on the revealed dependencies are given in the text. Abbreviations: AC—acylcarnitines; FC—functional class; SCOS—clinical status assessment scale; CHF—chronic heart failure; CHD—coronary heart disease.

An intergroup comparison of AC levels was performed for this cohort of patients, with the grouping characteristic of FC I–IV (Figure 5). Significant group-wide differences were shown mainly for long- and medium-chain ACs (C10–18), and differences were also found in the levels of short-chain propionyl- (C3), hydroxyisovaleryl- (C5-OH) and adipoylcarnitines (C6-DC). By posterior analysis (Figure 3), significant differences were determined between groups I + II and III FCs in the levels of hydroxyhexadecenoylcarnitine (C16:1-OH); between groups I + II and IV FCs in the levels of hydroxyisovaleryl- (C5-OH), decadienoyl- (C10:2) and hydroxyhexadecenoylcarnitine (C16:1-OH). FC III and IV differed in the levels of propionyl- (C3), decenoyl- (C10:1), dodecadienoyl- (C12:1) and tetradecadienoylcarnitines (C14:2). We note that in all cases, there was a significant increase in ACs levels with increasing FC, except for C3 and C5-OH in the corresponding intergroup comparisons—their levels were higher at lower FCs. 

## 4. Discussion

ACs are of interest because in the field of metabolomic profiling, there is increasing evidence for the use of these metabolites as markers of CVD: insulin resistance [11], arterial hypertension [12], atrial fibrillation [13,14], CHD [15] and CHF [16,17,18]. In previous studies presented in scientific databases, we did not find a direct comparison of AC levels in patients with CHD and CHF of ischemic etiology (Table 5). The novelty of our work was the analysis of a wider range of MR parameters and functional parameters of the heart. In our comparative study, we hypothesized that changes in FA metabolism, which cause AC accumulation, may be associated with structural and functional parameters of myocardial remodeling, as well as with the clinical picture in patients with CHF of ischemic etiology and systole-diastolic dysfunction. We found an elevation of a number of ACs in both groups with significant differences between the groups of CHF complicated by the development of CHD, mainly in the levels of long- and medium-chain ACs (C10; C12; C14; C16; C18); we also found a difference in the level of short-chain butyrylcarnitine (C4). We note that a practically similar pool of AC is associated with remodeling parameters and clinical characteristics in patients with CHF of ischemic etiology. The obtained correlations may indicate the connection of increased levels of AC with signs of decompensation of CHF in the large circulation circle and indirectly with congestion in the small circulation circle. 

In fundamental works devoted to the study of myocardial metabolism in CHF [16], it is noted that it is able to return to the genetic program of the embryo [23], existing in anaerobic conditions, and partially switch between the types of ATP production from FA oxidation to the oxidation of carbohydrates, i.e., glucose as the main substituting energy substrate, as well as intermediate products: lactate, ketones, etc. [24]. Thus, myocardium can exist for a long time under conditions of metabolic remodeling with the key purpose: to provide pumping function of the heart, primarily LV, as the most energy-consuming part of myocardium. However, with the progression of CHF, the formation of endothelial dysfunction, insulin resistance, excessive body weight and other metabolic disorders that develop not only in the heart but also in the vessels, the ability to utilize glucose decreases, and so does the oxidation of FA accompanied by the accumulation of AC [24,25,26]. 

Indeed, several studies have shown an association of AC with CHF and systolic dysfunction. In experimental works on mouse models, the authors describe these changes in the framework of desynchronization of glucose and FA oxidation in CMC of hypertrophied myocardium with inhibition of mitochondrial enzyme CPT-1/2 and PPARα, PGC1α receptors directly by AC excess, which leads to the limitation of β-oxidation and formation of lipotoxicity [27]. The authors who evaluated myocardial metabolism in the conditions of diabetes on the background of CHF development on animal models came to similar conclusions. They also note that the basis is metabolic remodeling determined by CMC rearrangement from β-oxidation of FA due to mitochondrial dysfunction to oxidation of glucose and ketone bodies in the cell cytoplasm [28]. 

The normal range of carnitine varies from 22 to 65 μM. The normal ranges of carnitine and its derivatives according to the Human Metabolomic Database (HMDB) are presented in Table 4 (Table 4). Different studies showed increases in ACs with different carbon chain lengths, circulating unesterified carnitine (C0) in patients with CHF and CHD, defining them as markers of both CMC dysfunction and chronic inflammation observed in both diseases (Table 5). Other teams have noted a consistent opposite effect, i.e., a decrease in circulating AC levels after restoration of LV pump function, by mechanical LV support, in people with aortic stenosis and CHF [29]. The results of our study show the absence of correlation of increased AC levels with both known types of LV remodeling and its volume-dimensional indices. The revealed correlations, on the contrary, are associated with remodeling of the least energy-consuming parts of the heart—atria, RV. Our data on the association of AC with the signs of CHF decompensation and parameters reflecting the circulation overload suggest the presence of possible other causes of AC level increase. Some authors consider the role of intestinal microbiota as a source of circulating ACs, since bacterial overgrowth, inflammation, and increased permeability of the intestinal wall are characteristic of congestive CHF [30,31,32]. An additional risk factor is also low physical activity of patients with CHF, leading to poor consumption of medium- and long-chain ACs not only in myocardium, but also in peripheral, skeletal, muscles and, as a consequence, increased plasma concentrations of these AC fractions [33]. The above-mentioned metabolic disorders are characteristic stigmas of patients with CHF and our cohort.

However, if we refer to the basics of hemodynamics, it is known that myocardium of atria and RV is not able to resist prolonged overload [34], which in patients with decompensated CHF is determined by increased venous pressure in both circles. It is probable that these features lead to more rapid metabolic remodeling of myocardium of the above parts against the background of greater myocardial stress than in LV and, as a result, accumulation of underoxidized medium- and long-chain ACs. This is confirmed not only by studies in which we evaluated the relationship of this AC fraction with remodeling of myocardium of RA and RV on the background of pre- and postcapillary pulmonary hypertension [35,36], but also by a clear correlation of this fraction with the increase of pulmonary artery pressure in our work. 

Given the proven role of excessive accumulation of intracellular and circulating ACs in aggravation of cytotoxicity and, at the same time, the ambiguity of mechanisms explaining their accumulation in patients with CHF of ischemic etiology, it is necessary to conduct further prospective studies. It is important to study the peculiarities of metabolism and remodeling of both “right” heart departments and large vessels, such as PA and VCI. 

Such observations suggest the possibilities of therapy with carnitine in case of its deficiency. There are works where the effectiveness of supplements containing L-carnitine and its derivatives was analyzed in relation to patients with various CVD and positive effects, such as an increase in LVEF, a decrease in LV volumes, and a decrease in serum levels of sodium-uretic peptides. However, there are no studies among them that describe carnitine therapy in patients with LV and LVEF < 50%. 

Dambrova M. and co-authors reviewed existing data by 2022 on the effect of L-carnitine on the course of cardiovascular diseases: CHD, CHF, hypertension. The intended effect of these supplements is to increase the binding of fatty acids (acyls) and enhance their transport to the mitochondria for oxidative phosphorylation, thereby reducing the pool of circulating cytotoxic acylcarnitines [37]. But it is not clear how the use of these supplements can affect the health of patients whose levels of carnitine and its derivatives are increased initially, as in our study. Moreover, it was shown that L-carnitine supplementation was associated with a greater progression of carotid stenosis compared to placebo in patients with risk factors [38]. Therefore, carnitine therapy remains controversial and requires additional clarifying studies. Nevertheless, the lack of evidence (11 publications in total, where the effect of additives on various CVD was evaluated) opens the question of the effectiveness of therapy with this class of biological substances [39,40].

According to the results of our study, there are differences between the comparison groups in the levels of medium- and long-chain acylcarnitines in favor of relatively high levels of these fractions in patients with HF. At the same time, 50% of this cohort have AC values below the given reference values. In this regard, it is difficult to determine the clinical applicability of the data obtained.

In addition, the question remains unresolved as to whether the levels of circulating ACs change with HFpEF or HF of non-ischemic etiology. Further studies of AC profiles in various HF phenotypes are needed to continue the search for new approaches to targeted therapy of rapidly spreading pathology.

Limitations of our study included insufficient study power, different volumes of comparable samples, and the identification of plasma ACs in patients with comorbidities and multidrug therapy, which together may influence plasma metabolite levels. An improved epidemiologic design of follow-up studies, comparable samples of sufficient size, and a targeting study of CMC metabolites in human cardiac tissue may probably provide answers to outstanding questions on this topic.

## 5. Conclusions

Heart failure of ischemic etiology remains a key problem in cardiology, and episodes of decompensation and progressive course of the disease, as well as myocardial remodeling—a significant burden for patients. At the same time, there is no doubt about the reversibility of cardiac remodeling and slowing of CHF progression with adequate drug therapy. According to the results of the study, despite the excess of carnitine levels, in patients with CHF of ischemic etiology and LVEF < 50% due to accelerated beta-oxidation of lipids, there is a deficiency of carnitine derivates with medium and long chain lengths. However, our work has shown that AC levels are associated not so much with cardiac remodeling in pure form but with many factors determining the severity of the course of CHF—congestion in both circulatory circles and overload of the right heart. All these results may have clinical significance if future studies confirm similar associations with changes in the profile of carnitine and derivatives. This, in turn, may allow further evaluation of the effectiveness of carnitine derivative deficiency therapy.

## Figures and Tables

**Table 1:** General characteristics of the comparison groups.

Characteristics	CHF-CHD Group (n = 79)	CHD Group (n = 19)	*p*-Value
Gender, male	56 (70.9%)	10 (52.6%)	0.13
Age, years	68.2 ± 7.2	66.5 ± 9	0.38
BMI, kg/m^2^	30.7 [27.6; 34.6]	30.6 ± 5.8	0.65
NT-proBNP, pg/mL	2767.5 [1392.4; 3562.5]	140 [54; 176]	<0.05

Me [Q1; Q3]—median (Me) and interquartile range [Q1; Q3]; M ± SD—the mean (M), standard deviation (SD). Abbreviations: CHF—chronic heart failure; CHD—coronary heart disease; BMI—body mass index.

**Table 2:** Co-morbidities in patients in the comparison groups.

Characteristics	CHF-CHD Group (n = 79)		CHD Group (n = 19)		
	Number of Patients	% of Total	Number of Patients	% of Total	*p*-Value
**History of MI**	58	73	8	27.8	0.009
**Rhythm disturbances:** **AF/AF:** **Paroxysmal form** **Persistent form** **Permanent form** **Pacemaker rhythm**	49 14 15 20 4	62 18 19 25 5	5 5 - - -	26.3 26.3 - - -	0.005
**Hypertension**	79	100	19	100	-
**Stroke**	9	11.4	3	15.8	0.6
**CKD, eGFR <60 mL/min/1.73 m^2^**	50	63.3	7	36.8	0.42
**Glucose disorders** **DM** **IGT**	38 20	48.1 25.3	9 2	47.4 10.5	0.25
**Dyslipidemia**	57	72.1	17	89.5	0.11
**Remodeling type according** **to LVMI/RWT:** **Normal geometry** **Concentric remodeling** **Concentric hypertrophy** **Eccentric hypertrophy**	7 10 31 31	9 12.6 39.2 39.2	4 12 3 -	21.1 63.1 15.8 -	<0.001

Abbreviations: CHF—chronic heart failure; CHD—coronary heart disease; MI—myocardial infarction; AF—atrial fibrillation; AF—atrial flutter; CKD—chronic kidney disease; eGFR—estimated glomerular filtration rate according to MDRD calculation formula; DM—diabetes mellitus; IGT—impaired glucose tolerance; RWT—relative wall thickness index; LVMI—left ventricular myocardial mass index.

**Table 3:** Descriptive and comparative characteristics of echocardiographic parameters in patients with CHF of ischemic etiology and patients with CHD without signs of CHF.

Heart Chamber	Parameters	Reference	CHF-CHD Group (n = 79)	CHD Group (n = 19)	*p*-Value
**Left ventricle (LV)**	EDD, mm	≤58.4 (m), ≤52.2 (f)	55 ± 7	47 ± 3.1	<0.001
	ESD, mm	≤39.8 (m), ≤34.8 (f)	44 ± 7.6	31 ± 3.6	<0.001
	EDV index, mL/m^2^	<75 (m), <62 (f)	69 [56; 86]	47.9 ± 9	<0.001
	ESV, mL	<58 (m), <49 (f)	92 [64; 117]	40.2 ± 10.9	<0.001
	SV index, mL/min/m^2^	>35	27.4 ± 7.6	26.9 [23.6; 30.9]	0.25
	Ejection fraction (Biplane), %	>52 (m), >54 (f)	37 [31; 45]	58 [55; 60]	<0.001
	IVS, mm	≤10 (m), ≤9 (f)	12 [11; 13]	10.9 ± 1.8	0.02
	LVPW, mm	≤10 (m), ≤9 (f)	11 [10; 12]	10.6 ± 1.4	0.24
	LVMI, g/m^2^	≤95 (g), ≤115 (m)	129.3 [108.4; 142.6]	93.2 ± 15.7	<0.001
**Left atrium (LA)**	mm	≤40 (m), ≤38 (f)	46 [44; 50]	37.8 ± 5.6	<0.001
	LA volume index, mL/m^2^	≤34	44 [38; 52]	28.2 [23.2; 30.9]	<0.001
**Right ventricle (RV)**	Basal diameter, mm	<42	39 [36; 44]	34.2 ± 5.6	<0.001
	Free wall thickness, mm	<5	4 [4; 4]	4 [3; 4]	0.43
**Right atrium (RA)**	RA volume index, mL/m^2^	<30 (m), <28 (f).	33 [26; 41]	22.2 [17.2; 24.9]	<0.001
**Aortic dimensions**	Indexed size of Valsalva sinus, mm/m^2^	≤19 (g), ≤20 (f)	16.5 ± 2	16.7 [14.5; 17.6]	0.91
	Indexed proximal ascending aorta size, mm/m^2^	≤17 (g), ≤19 (f)	15.9 ± 2.1	15.9 [13.8;17]	0.65
**Inferior vena cava (IVC)**	mm	<21	21 ± 4	20 ± 2.2	0.33
**Pulmonary artery (PA)**	PA size, mm	<25	24 [23; 25]	23 [22; 23]	<0.001
	Estimated systolic pressure in LA, mm Hg.	<31	42 [31; 54]	25 [22; 29]	<0.001

Me [Q1; Q3]—median (Me) and interquartile range [Q1; Q3]; M ± SD—the mean (M), standard deviation (SD). Abbreviations: CHF—chronic heart failure; CHD—ischemic heart disease; EDD—end-diastolic dimension; EDS, end-systolic dimension; EDV—end-diastolic volume; ESV—end-systolic volume; SV—stroke volume; IVS—interventricular septum; PW—posterior wall of the LV; LVM—myocardial mass; LVMI—LV myocardial mass index.

**Table 4:** Intergroup comparison of plasma acylcarnitine levels.

Name of Acylcarnitines	Abbreviations	Class	CHF-CHD Group (N = 79) (Μm)	CHD Group (N = 19) (Μm)	*p*-Value	Normal Range (From HMDB Database)		Rates of LVEF < 50% in Patients with Deficit of Carnitine and Acs	Mean Value in Relation to The Limits of The Range: CHF-CHD Group; CHD Group
						Suggested Range (μM)	Reference	Number and %	
**Carnitine**	C0	Unesterified	83.6 [48; 133]	104.9 [67.2; 155.3]	0.26	22.19–37.29	http://www.ncbi.nlm.nih.gov/pubmed/21359215, accessed on 15 August 2023	5 (5.1%)	CHF-CHD: 2.2 times higher than the upper limit; CHD: 2.8 times higher than the upper limit
						25.4–54.1	15519880		CHF-CHD: 1.5 times higher than the upper limit; CHD: 1.9 times higher than the upper limit
						32.8–43.6			CHF-CHD: 1.9 times higher than the upper limit; CHD: 2.4 times higher than the upper limit
						33.6–53.54	http://www.ncbi.nlm.nih.gov/pubmed/26010610, accessed on 15 August 2023		CHF-CHD: 1.6 times higher than the upper limit; CHD: 2 times higher than the upper limit
						26.08–52.58	26010610		CHF-CHD: 1.05 times higher than the upper limit; CHD: 1.33 times higher than the upper limit
						19.0–65.0	http://metabolomicscentre.ca/, accessed on 15 August 2023		CHF-CHD: 1.3 times higher than the upper limit; CHD: 1.6 times higher than the upper limit
						28.3–42.3	http://www.ncbi.nlm.nih.gov/pubmed/28278231, accessed on 15 August 2023		CHF-CHD: 2 times higher than the upper limit; CHD: 2.5 times higher than the upper limit
						34.1–57.3	21359215		CHF-CHD: 1.4 times higher than the upper limit; CHD: 1.8 times higher than the upper limit
**Acetylcarnitine**	C2	Short-chain	13.43 [9.1; 20.48]	11.93 [7.5; 19.7]	0.21	3.33–7.63	21359215	12 (12.2%)	CHF-CHD: 1.7 times higher than the upper limit; CHD: 1.56 times higher than the upper limit
						3.00–12.5	http://metabolomicscentre.ca/, accessed on 15 August 2023		CHF-CHD: 10.7 times higher than the upper limit; CHD: Close to the upper limit
						5.6–6.8 (male)	http://www.ncbi.nlm.nih.gov/pubmed/12905800, accessed on 15 August 2023		CHF-CHD: 2 times higher than the upper limit; CHD: 1.7 times higher than the upper limit
						5.0–6.4 (female)	12905800		CHF-CHD: 2.1 times higher than the upper limit; CHD: 1.9 times higher than the upper limit
						4.30–8.82	28278231		CHF-CHD: 1.5 times higher than the upper limit; CHD: 1.4 times higher than the upper limit
**Propionylcarnitine**	C3	Short-chain	0.6 [0.4; 0.79]	0.63 ± 0.27	0.96	0.26–0.46	21359215	20 (20.4%)	CHF-CHD: 1.3 times higher than the upper limit; CHD: 1.4 times higher than the upper limit
						0.379–0.421	16425363		CHF-CHD: 1.3 times higher than the upper limit; CHD: 1.4 times higher than the upper limit
						0.15–0.7	http://metabolomicscentre.ca/, accessed on 15 August 2023		CHF-CHD: Close to the upper limit; CHD: Close to the upper limit
						0.30–0.58	26010610		CHF-CHD: Close to the upper limit; CHD: Close to the upper limit
						0.24–0.44	http://www.ncbi.nlm.nih.gov/pubmed/28278231, accessed on 15 August 2023		CHF-CHD: 1.4 times higher than the upper limit; CHD: 1.4 times higher than the upper limit
**Butyrylcarnitine**	C4	Short-chain	0.175 [0.12; 0.22]	0.12 [0.11; 0.18]	0.036	0.10–0.42	21359215	12 (12.2%)	CHF-CHD: Close to the lower limit; CHD: Close to the lower limit
						0.10–0.45	http://metabolomicscentre.ca/, accessed on 15 August 2023		CHF-CHD: Close to the lower limit; CHD: Close to the lower limit
						0.254–0.28	http://www.ncbi.nlm.nih.gov/pubmed/16425363, accessed on 15 August 2023		CHF-CHD: 1.5 times lower than the lower limit; CHD: 2.1 times lower than the lower limit
**Tiglylcarnitine**	C5:1	Short-chain	0.013 [0.01; 0.02]	0.012 ± 0.005	0.43	0.04–0.06	21359215	75 (76.5%)	CHF-CHD: 3.1 times lower than the lower limit; CHD: 3.3 times lower than the lower limit
						0.04–0.08	Molecular You		CHF-CHD: 3.1 times lower than the lower limit; CHD: 3.3 times higher than the upper limit
**Isovalerylcarnitine**	C5	Short-chain	0.07 [0.05; 0.1]	0.07 [0.06; 0.08]	0.98	0.128–0.148	16425363	65 (66.3%)	CHF-CHD: 1.8 times lower than the lower limit; CHD: 1.8 times higher than the upper limit
**Hydroxyisovalerylcarnitine**	C5-OH	Short-chain	0.005 [0.003; 0.007]	0.004 [0.003; 0.005]	0.09	< 0.51	15505778	0 (0%)	CHF-CHD and CHD: Normal level
**Glutarylcarnitine**	C5-DC	Short-chain	0.1 [0.06; 0.14]	0.1 [0.08; 0.12]	1.00	0.015–0.04	Molecular You	74 (75.5%)	CHF-CHD and CHD: In 2.5 times higher than the upper limit
**Hexanoylcarnitine**	C6	Medium-chain	0.06 [0.04; 0.07]	0.047 [0.04; 0.06]	0.11	0.074–0.086	16425363	60 (61.2%)	CHF-CHD: 1.23 times lower than the lower limit; CHD: 1.6 times lower than the lower limit
						0.02–0.13	Molecular You		CHF-CHD: Close to mean of the range; CHD: Close to mean of the range
						0.04–0.08	28278231		CHF-CHD: Close to mean of the range; CHD: Close to the lower limit
**Adipoylcarnitine**	C6-DC	Medium-chain	0.015 [0.01; 0.02]	0.019 [0.01; 0.03]	0.19	-	-	-	-
**Octenoylcarnitine**	C8:1	Medium-chain	0.023 [0.016; 0.03]	0.017 [0.01; 0.04]	0.17	0.05–0.35	http://www.ncbi.nlm.nih.gov/pubmed/21359215, accessed on 15 August 2023	74 (75.5%)	CHF-CHD: 1.23 times lower than the lower limit; CHD: 3 times lower than the lower limit
**Octanoylcarnitine**	C8	Medium-chain	0.166 [0.12; 0.2]	0.15 [0.11; 0.19]	0.27	0.15–0.31	http://www.ncbi.nlm.nih.gov/pubmed/21359215, accessed on 15 August 2023	32 (32.6%)	CHF-CHD: Close to the lower limit; CHD: Close to the lower limit
						0.112–0.13	16425363		CHF-CHD: 1.3 times higher than the upper limit; CHD: 1.2 times higher than the upper limit
						0.17–0.5	http://metabolomicscentre.ca/, accessed on 15 August 2023		CHF-CHD: Close to the lower limit; CHD: 1.13 times lower than the lower limit
						0.15–0.27	http://www.ncbi.nlm.nih.gov/pubmed/26010610, accessed on 15 August 2023		CHF-CHD: Close to the lower limit; CHD: Close to the lower limit
						0.11–0.25	http://www.ncbi.nlm.nih.gov/pubmed/28278231, accessed on 15 August 2023		CHF-CHD: Close to the lower limit; CHD: Close to the lower limit
						0.17–0.5	http://metabolomicscentre.ca/, accessed on 15 August 2023		CHF-CHD: Close to the lower limit; CHD: Close to the lower limit
**Decanoylcarnitine**	C10	Medium-chain	0.27 [0.2; 0.4]	0.27 ± 0.13	0.52	0.15–0.37	21359215	10 (10.2%)	CHF-CHD: Close to mean of the range; CHD: Close to mean of the range
						0.132–0.15	16425363		CHF-CHD: 1.8 times higher than the upper limit; CHD:In 1.8 times higher than the upper limit
						0.18–0.44	http://www.ncbi.nlm.nih.gov/pubmed/28278231, accessed on 15 August 2023		CHF-CHD: Close to mean of the range; CHD: Close to mean of the range
						0.16–0.55	http://metabolomicscentre.ca/, accessed on 15 August 2023		CHF-CHD: Close to mean of the range; CHD: Close to mean of the range
						0.14–0.32	http://www.ncbi.nlm.nih.gov/pubmed/26010610, accessed on 15 August 2023		CHF-CHD: Close to the upper limit; CHD: Close to the upper limit
**Decenoylcarnitine**	C10:1	Medium-chain	0.20 [0.15; 0.3]	0.17 [0.12; 0.19]	0.05	0.12–0.4	http://metabolomicscentre.ca/, accessed on 15 August 2023	8 (8.2%)	CHF-CHD: Close to the lower limit; CHD: Close to the lower limit
**Decadienoylcarnitine**	C10:2	Medium-chain	0.01 [0.01; 0.02]	0.009 [0.007; 0.014]	0.01	-	-	-	-
**Dodecanoylcarnitine**	C12	Medium-chain	0.08 [0.05; 0.1]	0.06 [0.047; 0.07]	0.03	0.048–0.056	http://www.ncbi.nlm.nih.gov/pubmed/16425363, accessed on 15 August 2023	64 (65.3%)	CHF-CHD: 1.4 times higher than the upper limit; CHD: Close to the upper limit
						0.07–0.13	21359215		CHF-CHD: Close to the lower limit; CHD: Close to the lower limit
						0.057–0.19	Molecular You		CHF-CHD: Close to the lower limit; CHD: Close to the lower limit
						0.048–0.056	16425363		CHF-CHD: 1.4 times higher than the upper limit; CHD: Close to the upper limit
						0.057–0.19	Molecular You		CHF-CHD: Close to the lower limit; CHD: Close to the lower limit
						0.005–0.069	9034211		CHF-CHD: 1.2 times higher than the upper limit; CHD: Close to the upper limit
**Dodecenoylcarnitine**	C12:1	Medium-chain	0.04 [0.03; 0.06]	0.032 [0.027; 0.045]	0.009	0.1–0.4	Molecular You	5 (5.1%)	CHF-CHD: 2.5 times lower than the lower limit; CHD: 3.1 times lower than the lower limit
**Tetradecanoylcarnitine**	C14	Long-chain	0.03 [0.02; 0.04]	0.02 ± 0.007	0.01	0.03–0.05	http://www.ncbi.nlm.nih.gov/pubmed/21359215, accessed on 15 August 2023	40 (40.8%)	CHF-CHD: Close to the lower limit; CHD: 1.5 times lower than the lower limit
**Tetradecenoylcarnitine**	C14:1	Long-chain	0.053 [0.04; 0.08]	0.036 [0.024; 0.045]	0.001	0.03–0.09	21359215	11 (11.2%)	CHF-CHD: Close to mean of the range; CHD: Close to the lower limit
						0.02–0.24	Molecular You	3 (3%)	CHF-CHD: Close to the lower limit; CHD: Close to the lower limit
**Tetradecadienoylcarnitine**	C14:2	Long-chain	0.029 [0.02; 0.04]	0.018 [0.01; 0.02]	<0.001	-	-	-	-
**Hydroxytetradecanoylcarnitine**	C14-OH	Long-chain	0.0005 [0.0003; 0.001]	0.0004 [0.0003; 0.0005]	0.18	0.015–0.03	Molecular You	79 (80.6%)	CHF-CHD: 30 times lower than the lower limit; CHD: 37.5 times lower than the lower limit
**Palmitoylcarnitine**	C16	Long-chain	0.11 [0.09; 0.13]	0.095 ± 0.02	0.04	0.107–1.119	http://www.ncbi.nlm.nih.gov/pubmed/16425363, accessed on 15 August 2023	38 (38.7%)	CHF-CHD: Close to the lower limit; CHD: 1.13 times lower than the lower limit
**Hexadecenoylcarnitine**	C16:1	Long-chain	0.023 [0.02; 0.03]	0.018 ± 0.008	0.002	0.01–0.06	https://molecularyou.com/, accessed on 15 August 2023	3 (3%)	CHF-CHD: Close to the lower limit; CHD: Close to the lower limit
						0.02–0.04	http://www.ncbi.nlm.nih.gov/pubmed/21359215, accessed on 15 August 2023		CHF-CHD: Close to the lower limit; CHD: Close to the lower limit
**Hydroxyhexadecenoylcarnitine**	C16:1-OH	Long-chain	0.0017 [0.0007; 0.003]	0.001 [0.0006; 0.002]	0.33	-	-	-	-
**Hydroxyhexadecanoylcarnitine**	C16-OH	Long-chain	0.042 [0.03; 0.05]	0.038 [0.034; 0.06]	0.76	0.005–0.02	Molecular You	0 (0%)	CHF-CHD: 2.1 times higher than the upper limit; CHD: 1.9 times higher than the upper limit
**Stearoylcarnitine**	C18	Long-chain	0.03 [0.02; 0.04]	0.03 ± 0.009	0.91	0.03–0.05	21359215	37 (37.7%)	CHF-CHD: Close to the lower limit; CHD: Close to the lower limit
**Oleoylcarnitine**	C18:1	Long-chain	0.073 [0.06; 0.09]	0.055 ± 0.017	0.001	0.04–0.21	Molecular You	9 (9.2%)	CHF-CHD: Close to the lower limit; CHD: Close to the lower limit
**Hydroxyoctadecenoylcarnitine**	C18:1-OH	Long-chain	0.0003 [0.0002; 0.0006]	0.0003 [0.0001; 0.0005]	0.49	0.000–0.023	Molecular You	0 (0%)	CHF-CHD: Close to the lower limit; CHD: Close to the lower limit
**Linoleoylcarnitine**	C18:2	Long-chain	0.043 [0.03; 0.06]	0.03 ± 0.01	<0.001	0.03–0.09	21359215	20 (20.4%)	CHF-CHD: Close to the lower limit; CHD: Close to the lower limit
**Hydroxyoctadecanoylcarnitine**	C18-OH	Long-chain	0.0007 [0.0006; 0.001]	0.0008 ± 0.0004	0.38	-	-	-	-

Me [Q1; Q3]—median (Me) and interquartile range [Q1; Q3]; M ± SD—the mean (M), standard deviation (SD). Abbreviations: CHF—chronic heart failure; CHD—coronary heart disease.

**Table 5:** Acylcarnitine changes in previous studies.

Title	Demographic Characteristics	CHD	HF	Changes in Acylcarnitines
**Karagiannidis, E. (2022).** **[19]**	N = 316 patients, mean age 67 ± 11 years old; 70.3% male	+	−	Increasing levels of ceramide ratio C24:1/C24:0, acylcarnitine ratio C4/C18:2.
**Deda, O. (2022). [15]**	N = 958 serum samples	+	−	Elevation of short-chain acylcarnitine C2, C4, C5 and C6 levels. Long-chain acylcarnitines C16, C18:1, and C18:2 were higher in Stable angina compared to STEMI. Ratio C4/C18:2 is useful for the prediction of CAD severity
**Gander, J. (2021).** **[20]**	N = 116, mean age 70.8 ± 8.7 years old; 65% male	+	−	Circulating medium- and long-chain acylcarnitines, especially C6:0, C8:0, C8:1, C12:1, C14:1, C16:0, C16:1, C18:1, and C20:4, were found to be elevated in CAD patients.
**Chen, W.S. (2020).** **[21]**	N = 79 HF patients hospitalized because of acute decompensation with a left ventricular ejection fraction (LVEF) < 40%; mean age 61.5 ± 13.0 years old; 64.6% male	−	+	Nine acylcarnitines could discriminate the IMP group from the NIMP group, including three long-chain (C18:1, C16, and C16:1) and six short-chain acylcarnitines (C5, C5-OH, C4, C4:1-DC, C3, and C2).
**Selvaraj, S. (2022).** **[22]**	N = 234 DEFINE-HF patients, mean age 62 ± 11 years old; 75% male	−	+	Changes in LCAC/dicarboxylated LCAC were positively associated with change in NT-proBNP, whereas changes in proline and histidine (factor 10) were negatively associated with changes in NT-proBNP.

Abbreviations: CHD—coronary heart disease; HF—heart failure; STEMI—ST-elevation myocardial infarction; LVEF—left ventricular ejection fraction; IMP/NIMP—“improved” and “non-improved” groups of HF-patients defined by the changes in LVEF from baseline to 12 months after discharge in Chen, W.S. and coathors’ study; LCAC—long-chained acylcarnitines.

## Data Availability

The data presented in this study are available on request from the corresponding author. The data are not publicly available because some of the data set will be used for further research.
